# “Immune Boosting” in the time of COVID: selling immunity on Instagram

**DOI:** 10.1186/s13223-020-00474-6

**Published:** 2020-09-03

**Authors:** Darren N. Wagner, Alessandro R. Marcon, Timothy Caulfield

**Affiliations:** Health Law Institute, Faculty of Law, Law Centre, University of Alberta, Edmonton, AB T6G 2H5 Canada

**Keywords:** Immune boosting, Social media, COVID-19, Instagram, Immunity

## Abstract

“Immune boosting” is a trending topic during the COVID-19 pandemic. The concept of “immune boosting” is scientifically misleading and often used to market unproven products and therapies. This paper presents an analysis of popular immune-boosting posts from Instagram. Of the sampled posts, all promoted “immune boosting” as beneficial, nearly all involved commercial interests, and many used scientific and medical rhetoric in their messaging.

## Introduction

“Immune boosting” is a trending topic correlated with the coronavirus pandemic, appearing alongside numerous speculative cures, treatments, and preventative strategies [1–4]. An analysis of Google Trends, for example, shows that the phrases “immune boost” and “immune boosting” saw a large increase in February 2020, around the same time concerns around the virus intensified. Further, from April 15th, 2020 to May 15th 2020, the popular hashtag #immunebooster increased on Instagram posts by over 46%. The idea of boosting one’s immunity, however, is misleading and scientifically inaccurate [2]. There is no current evidence that any product or practice will contribute to enhanced “immune boosting” protection against COVID-19 [5]. This lack of evidence has not stopped wellness gurus, celebrities, and commercial entities from propagating notions of boosting immunity, and messaging of this nature is readily found connected to online portrayals of COVID-19 in the popular press. With the abundance of misinformation circulating online [6], this research provides a sense of how immune-boosting discourse is presented on Instagram, one of the world’s largest social media platforms.

Instagram, the photo- and video-sharing social networking service owned by Facebook, Inc., has over 500 million users active daily [7]. Hashtags (e.g. #immunebooster) allow Instagram users to categorize their posts and to search for topic-related content. Hashtagged content can create popular trends, which promote perspectives and drive narratives [8]. Research has shown that social media sites like Instagram are becoming increasingly commercialized, generating profits for both companies and users. Indeed, Instagram has been described as a key player in “the attention economy” [9], where social media platforms strive to keep audiences logged in and active. It is reported that over 200 million Instagram users visit at least one business profile daily [7].

Food, fitness, and health are among Instagram’s top themes [10] and Instagram content has been associated with both positive and negative health outcomes. For example, the platform has been used to create supportive networks for public health initiatives like breastfeeding [11]. But it is also associated with problematic health behaviours, including the promotion of potentially harmful substances [12, 13] and body image and eating disorder issues [14–16].

This study presents a snapshot portrayal of Instagram posts using #immunebooster. The current interest in the scientifically questionable concept of “immune boosting” may lead to the spread of inaccurate information about immunity that could instil a false sense of security leading to higher-risk behaviours. The spread of this type of misinformation may also lead to financial loss and greater confusion about how individuals can best to respond to the pandemic [17].

## Methods

During a period of heightened media attention around COVID-19, we searched for “#immunebooster” on Instagram once a day, for 1 week (May 4–10th, 2020). On each day, we took screenshots of the first 10 “Top posts,” which display popular trending content as determined by Instagram’s algorithm [18]. The objective was not to summarize the totality of #immunebooster discourse on Instagram, as generated by all users, but rather to provide a snapshot of the commonly shared, salient trends evident among popular posts. In other words, we were trying to capture what was most commonly seen by the general public searching for #immunebooster.

We captured a screenshot of each #immunebooster post, its caption, and tapped on the posted image to capture all visible tags. If the poster had loaded the first comment with hashtags, as is common practice, we also captured that comment. We did not capture other user comments, as user discussions fell outside the purview of this study. Additionally, on 2 days of the week (7 and 8 May 7th and 8th, 2020), we took screenshots of all #immunebooster “stories.” We collected relevant metadata, such as the number of followers for each account and the number of likes for each post. To protect the privacy of the users, all usernames were removed from the finalized dataset, and no usernames appear in these findings.

We performed a content analysis on the posts, making use of both inductive and deductive methods [19]. This design involved building an initial coding frame to survey the data, and then modifying this frame by conducting an overview analysis of the posts. We observed, for example, a substantial presence of commercial activity and, as a result, incorporated these elements into the frame. Combining the images in the post and the text in the caption, we categorized the importance of “immune boosting” in each post as either central or peripheral. A post was categorized as central if the image or caption explicitly addressed “immune boosting.” A post was labelled as peripheral if “immune boosting” was merely mentioned or tangential to the post’s core message, such as a standalone hashtag. We assessed if and how “immune boosting” was portrayed as beneficial, or whether the concept was questioned or critiqued. We also detailed the commercial aspects present. See the complete coding frame made available in Additional file 1. The data was first analyzed by one coder and then reviewed by a second. We noted all discrepancies and resolved disagreements by consensus-reaching sessions.

## Results

Our daily collection of top posts over a week amounted to 28 samples (n = 28) from 26 unique accounts, following the removal of duplicate content appearing on multiple days. The average number of days for a post to remain in the top 10 was 2.42 (see Table 1). These 28 top posts included 55 tags on images, more than 17,000 likes, 539 hashtags (413 unique; average of 19.25 hashtags per post), 1 URL, and 17 unique @s directed to other Instagram accounts. Collectively, the 26 account holders of these top posts tally more than 500,000 followers, demonstrating considerable influence among some Instagram users.

**Table 1:** Complete coding analysis of the unique top Instagram posts using #immunebooster over one week in May 2020 (n = 28)

Criteria	Totals for sample of 28 posts	Averages	
		Percentages	Mean
Commercial accounts	27	96.43%	–
Tags on images	55	–	1.96
“Likes”	17,333	–	619
Hashtags (#)	538	–	19.21
Unique mentions (@)	21	–	0.75
URLs	1	–	0.04
Listed paid promotions	3	10.71%	–
Followers	532,538	–	19,019
Days post remained in top 10	68	–	2.43
Immune boosting as central theme	17	60.71%	–
Portrayed immune boosting as beneficial	28	100%	–
Including critiques of immune boosting	0	0%	–
Reference to scientific or medical authority	8	28.57%	–
Tags linking to companies	51	–	1.82
@’s to companies	21	–	0.75
Unique hashtags	413	–	14.75
URLs belonging to companies	1	–	0.04
Unique companies tagged and mentioned	62	–	2.21
Companies distinct from account holder	53		1.89
Mentioning or tagging companies	21	75.00%	–

Types of companies	Number of type	Percentage of total
Food (ingredients and cooking)	34	33.33%
General health and wellness	13	12.75%
Clothing, fashion and accessories	13	12.75%
Exercise advice and products	9	8.82%
Beauty products and advice	9	8.82%
Nutrition supplements	8	7.84%
Essential oils	4	3.92%
Home décor and furnishings	4	3.92%
Self-help and self-improvement	3	2.94%
Travel	2	1.96%
Technology	1	0.98%
Brand management	1	0.98%
Non-government organization (humanitarian)	1	0.98%
TOTAL	102	

We found 17 of the 28 (61%) posts feature “immune boosting” as a central idea and 11 posts as peripheral. All the posts portray “immune boosting” as beneficial. Most posts portray or intimate a general benefit to “immune boosting.” Specific benefits are associated with improved mood, anti-inflammation, increased metabolism, disease prevention, personal protection, gut health, better cognition, and skin care. None of the posts critique or question the value or validity of “immune boosting” in any way. Seven posts refer or appeal to scientific or medical authorities, including dietitians, nutritionists, doctors, and experts. One post mentions scientific research or evidence, which, in this instance, was a single unsubstantiated reference to “clinical studies.” 8 posts refer to COVID-19 through such phrases and hashtags as “Now more than ever it’s important for us to boost our immunity,” “#lockdowncooking,” and “#quarantineandchill.”

Of the 26 unique accounts sampled, 25 were commercial accounts, which we defined as any account that sells or advertises commercial products or services. This definition includes “influencer” accounts that advertise other people’s or companies’ products or services. Three posts are listed as paid promotions. We identified a total of 62 different companies tagged or mentioned in these posts, 53 of which are distinct from the accountholder. 75% of the posts tagged one or more companies. In order of decreasing frequency, the focus of these companies are related to food (ingredients and cooking); general health and wellness; clothing, fashion, and accessories; exercise advice and products; beauty products and advice; nutrition supplements; essential oils; home décor and furnishings; self-help and self-improvement; travel; technology; brand management; and a humanitarian non-government organization.

About 30% of the posts appeal to some form of medical or scientific authority and several have text suggesting the rhetoric of scientific evidence, which is “text that draws on scientific sounding language in order to create a veneer of legitimacy” [20]. For instance, post 13 recommends a dinner recipe that “helps boost your Microbiome and immunity.” That post was made by a self-identified general practitioner and health journalist who sells a series of diet and recipe books. Post 5 featured a “registered nutritionist, author, media regular, and mom” offering some tips “to boost your #immunity,” which entailed taking their brand of supplement and probiotic products. Another example using scientific rhetoric is Post 15, from the account of a personal trainer and “Creative Brand Consultant” who sells protein bars. The advertised product in Post 15 is a supplement, which is described as “a unique formula made with clinically-studied microbiome strains” and containing “organic ashwagandha- a plant known for its adoptogenic properties.” Post 9 includes a softer appeal to scientific knowledge from an influencer’s account, which advertises a variety of healthy food and supplement products with dedicated discount codes. This post has an image of a labelled jar of apple cider vinegar gummies held above a “loaded oatmeal bowl.” The theme is about healthy living and the caption connects the product to the COVID-19 pandemic: “Apple cider vinegar is known to improve digestion, detoxes the body, and provides immunity boosting which I think we could all use a little extra right now!”

Three accountholders included medical warnings concerning their immune-boosting products or services. The caption for post 21 has the following bolded disclaimer: “The Content is not intended to be a substitute for professional medical advice, diagnosis, or treatment. Always seek the advice of your physician or other qualified health provider with any questions you may have regarding a medical condition. Never disregard professional medical advice or delay in seeking it.” This emphatic deference to medical expertise contrasts with another post’s warning. Post 6 lists numerous contraindications and cautions for an “immunity booster pose.” The caption suggests the yoga pose “powers our immunity by stimulating the digestive system and stimulate the thymus, an organ located behind the chest bone that is responsible for the growth of T-cells.” Taken with the medical jargon, the warning in post 6 serves to legitimize the claim that the pose offers real and substantial immunity benefit.

Most posts expressly or implicitly advertised products or services as “immune boosting.” The image in post 2, for example, has a woman holding a jar of juice and is captioned as an “ORGANIC IMMUNE BOOSTING TONIC” comprised of “Naturally FRESH homemade immune booster.” The accompanying description claims this “vitamin powered” tonic is anti-inflammatory, antioxidant, antibacterial, and anti-fungal and can fight infections, prevent asthma, boost metabolism, aid “health cell growth,” and counter viral infections. The post also promotes the accountholder’s IGTV channel dedicated to selling the “immune boosting” tonic. Many commercial posts associated “immune boosting” with other popular health and lifestyle topics, including gut health, anti-inflammation, veganism, and “natural” foods and products. Post 21 comes from an account selling a line of essential-oil health and beauty products. The post has an infographic for “FOODS TO BOOST THE BODY IMMUNE SYSTEM,” which graphic is reminiscent of a nutrition guide. This post also includes the bolded disclaimer discussed above.

Some posts mentioned the COVID-19 pandemic in connection to their advertised products and services. Post 4 offered “a FREE 30-day #GetImmunityUpgraded challenge.” The caption promised that the advertised challenge, which includes workout routines, food recipes, and expert advice, will “protect you, your family, your friends and our world,” and ultimately “help flatten the curve.”

## Discussion

Our analysis shows that popular posts hashtagged with #immunebooster on Instagram are devoid of sound science and full of commercial content. Biomedical jargon and authoritative signaling are commonly used to give credibility to scientifically unsound ideas around boosting one’s immunity. In nearly every post commercial endeavours are highlighted implicitly and explicitly.

For the week we observed “top” #immunebooster content on Instagram, the concept of “immune boosting” was unequivocally portrayed as beneficial. Benefits included bettering mood and digestion, protection against a variety of infections and disorders, and improved skin and appearance. The most common actions to achieve an immune boost were food and diet related, while other actions included supplements, essential oils, exercises, sweating, probiotics, and cold showers. A few posts included cautions about their immune-boosting advice. However, many content creators signalled claims of biomedical authority figures or used scientific jargon to promote their products, services, and messages. Questions can be asked whether these claims to medical science provide credibility to the explicit and tacit health claims in these social media posts.

We observed widespread commercial activity in the data set. Indeed, most accounts used “immune boosting” for marketing products and services, as well as in promoting their own Instagram influencer presence. The hashtag #immunebooster, therefore, is not only a rhetorical tool used to promote health-related products and services, but is, in and of itself, a rhetorical product used to generate activity in the online attention economy [1, 9].

The harms of the immune-boosting trend on Instagram might not be immediately apparent or quantifiable. Our short survey had a limited sample size that and was focused more on qualitatively observations. It was found that the immune-boosting trend on Instagram promotes misleading information about immunity and advances products and services of no proven immunological benefit. In the case of “immune boosting” in the time of COVID-19, social media is promoting science-free content for commercial ends.

## Supplementary information

**Additional file 1.** Supplementary Material: Coding Frame.

## Data Availability

Available upon request to the corresponding author.
