# Supplementary material for: “Immune Boosting” in the time of COVID: selling immunity on Instagram
Source: Allergy Asthma Clin Immunol. 2020 Sep 3;16:76. doi: 10.1186/s13223-020-00474-6 (PMC7468087; doi:10.1186/s13223-020-00474-6)
Supplement: Supplementary file 1 — Additional file 1. Supplementary Material: Coding Frame. [file 13223_2020_474_MOESM1_ESM.docx]

**Additional Data**

Coding Frame for “Immune Boosting in the Time of COVID: Selling Immunity on Instagram”

*Data set: Daily search of “#immunebooster,” screen-capturing 10 posts appearing as “top” (10 chosen as appearing left to right, top to bottom). Two daily searches of all #immunebooster stories as they appeared on those days.*

1. METADATA and BASIC DESCRIPTION – *ALL “list” items to be separated with semi colons*
2. Image (primary content): Choose 1 of: Food, Outdoors, People, People with product, People outdoors, Product (containers, logo, etc.), Other [description fill in]
3. Commercial Account? (1 = yes)
4. Account holder's product or service# of tags on image, [fill in list of tags]
5. # of image likes
6. Included caption (Y/N)?
7. # of total hashtags, [fill in hashtags]
8. # of @’s in caption, [fill in @’s]
9. # of URLs in caption, [fill in URLs]
10. Is the post listed as paid promotion (Y/N)?
11. # of followers for account holder [fill in]
12. # of days each post is in the top 10 [fill in]
13. OVERALL MESSAGING (Image + Caption)
14. Is the idea of immune boosting a central theme of the post? (Central/Peripheral)
15. Is the idea of immune boosting portrayed as beneficial (Y/N)?
16. If Y, why/how beneficial? (i.e. general (aid, helps, etc.); improves mood, etc.) [list]
17. Are actions described to boost immune system (Y/N)?
18. If Y, which actions are listed? (i.e. food/drink intake, take product shown, etc.) [list]
19. Is the idea of immune boosting linked to COVID-19 either implicitly or explicitly? (Y/N) If yes, copy relevant text [i.e., “these times”, #COVID, #coronavirus, etc.]
20. Is the idea of immune boosting critiqued in any way (i.e. bunk, ineffective, noise, etc.)?
21. Are there any references to science, evidence, research or authority figures (i.e. scientists, doctors, etc.)? [If yes, list]
22. HASHTAG, TAG, URL, @ analysis
23. Calculate number of tags that link to companies (including products) for each post
24. Calculate number of @’s in captions that are to companies or products for each post
25. Calculate total number of unique hashtags appearing across all data sets
26. Calculate number of URLs that belong to companies and/or products
27. Number of unique companies tagged and mentioned
28. Number of companies distinct from account holder
29. Posts with one or more companies tagged
30. “Quotable Quotes”
